# Realizing the Ultralow Lattice Thermal Conductivity of Cu_3_SbSe_4_ Compound via Sulfur Alloying Effect

**DOI:** 10.3390/nano13192730

**Published:** 2023-10-08

**Authors:** Lijun Zhao, Haiwei Han, Zhengping Lu, Jian Yang, Xinmeng Wu, Bangzhi Ge, Lihua Yu, Zhongqi Shi, Abdulnasser M. Karami, Songtao Dong, Shahid Hussain, Guanjun Qiao, Junhua Xu

**Affiliations:** 1 School of Materials Science and Engineering, Jiangsu University of Science and Technology, Zhenjiang 212100, China; 2 School of Materials Science and Engineering, Jiangsu University, Zhenjiang 212013, China; 3 School of Materials Science and Engineering, Northwestern Polytechnical University, Xi’an 710072, China; 4 State Key Laboratory for Mechanical Behavior of Materials, Xi’an Jiaotong University, Xi’an 710049, China; 5 Department of Chemistry, College of Science, King Saud University, Riyadh 11451, Saudi Arabia

**Keywords:** Cu_3_SbSe_4_-based materials, solid solutions, S alloying, point defect, thermoelectric properties

## Abstract

Cu_3_SbSe_4_ is a potential p-type thermoelectric material, distinguished by its earth-abundant, inexpensive, innocuous, and environmentally friendly components. Nonetheless, the thermoelectric performance is poor and remains subpar. Herein, the electrical and thermal transport properties of Cu_3_SbSe_4_ were synergistically optimized by S alloying. Firstly, S alloying widened the band gap, effectively alleviating the bipolar effect. Additionally, the substitution of S in the lattice significantly increased the carrier effective mass, leading to a large Seebeck coefficient of ~730 μVK^−1^. Moreover, S alloying yielded point defect and Umklapp scattering to significantly depress the lattice thermal conductivity, and thus brought about an ultralow *κ*_lat_ ~0.50 Wm^−1^K^−1^ at 673 K in the solid solution. Consequently, multiple effects induced by S alloying enhanced the thermoelectric performance of the Cu_3_SbSe_4_-Cu_3_SbS_4_ solid solution, resulting in a maximum ZT value of ~0.72 at 673 K for the Cu_3_SbSe_2.8_S_1.2_ sample, which was ~44% higher than that of pristine Cu_3_SbSe_4_. This work offers direction on improving the comprehensive TE in solid solutions via elemental alloying.

## 1. Introduction

Thermoelectric (TE) technology has the capability to directly and reversibly convert heat into electricity, making it a promising source of clean energy. It plays a significant role in addressing the challenges posed by the energy and environmental crises [1,2,3]. Numerous TE materials are currently under exploration for power generation and solid-state cooling applications, leveraging the Seebeck and Peltier effects, respectively [4], such as skutterudites [5], half-Heusler compounds [6], Zintl phases [7], chalcogenides [8], oxides [9,10], and high-entropy alloys [11]. Commonly, the conversion efficiency of TE materials is assessed using the dimensionless figure of merit, ZT = *S*^2^*σT*/*κ*, where *S*, *σ*, *T*, and *κ* stand for the Seebeck coefficient, electrical conductivity, absolute temperature in Kelvin, and total thermal conductivity (comprising lattice part *κ*_lat_ and electronic part *κ*_ele_), respectively [12,13]. Actually, achieving high conversion efficiency (η) necessitates a higher power factor (PF = *S*^2^*σ*) and/or lower *κ* [14,15,16,17,18,19,20]. Unfortunately, it is difficult to simultaneously optimize the *S*, *σ*, and *κ*_ele_ in the given TE material due to their strong coupling effects [12,21]. Nevertheless, *κ*_lat_ stands as the sole independently regulated TE parameter, leading to extensive research over the last two decades [16,17,19].

Copper-based chalcogenides have garnered significant attention because of their relatively favorable electrical transport and low thermal transport properties [22,23,24,25]. In addition, thermoelectric minerals like germanites, colusites, tetrahedrites, and other materials also have rather high ZT values [26,27,28]. Among them, the Cu_3_SbSe_4_ compound is a p-type semiconductor, featuring a narrow band gap of ~0.29 eV [29,30]. More importantly, its components are earth-abundant, inexpensive, non-toxic, and environmentally friendly [31,32]. However, its high *κ* and low *σ*, stemming from low carrier concentration and mobility, present challenges that hinder its practical use. Extensive efforts have been implemented to enhance the TE performance of Cu_3_SbSe_4_, including elemental doping [33,34,35,36,37], band engineering [38,39,40], and nanostructure modification [41,42]. These approaches have potential in improving the carrier concentration of (*n*), *S*, or *κ*_lat_, and thus leading to an appealing figure of merit. Although high *n* can enhance *σ*, it has a negative impact on *S* and result in an increase in *κ*_ele_. The TE performance of Cu_3_SbSe_4_ falls significantly short of that of Cu-based chalcogenides due to these two inherent issues. On one side, the narrow energy band gap of ~0.29 eV leads to bipolar diffusion, causing deterioration in electrical properties [29,30]. On the other side, the high thermal conductivity (*κ*_lat_) inherently arises from its composition comprising lightweight elements and a diamond-like structure [25]. In other words, optimizing carrier concentration alone proves challenging in further enhancing the TE performance.

The formation of a solid solution via elemental alloying is an effective strategy for depressing the *κ*_lat_ and thereby enhancing the TE performance. For example, Skoug et al. demonstrated that the substitution of Ge on Sn sites can lead to the formation of Cu_2_Sn_1−*x*_Ge*_x_*Se_3_ solid solutions, synergically optimizing the TE properties [43]. Jacob et al. reported that a high ZT_max_ value of ~0.42 was obtained in the Cu_2_Ge(S_1−*x*_Se*_x_*)_3_ system via Se alloying [44]. Wang et al. enhanced the TE properties of Cu_2_Ge(Se_1−*x*_Te*_x_*)_3_ by incorporating Te on the Se site, resulting in a ZT_max_ of ~0.55, which was 62% higher than that of the matrix [45]. The afore-mentioned research give us an idea that the Cu_3_Sb(Se_1−*x*_S*_x_*)_4_ solid solution is an effectively strategy for enhancing the thermoelectric performance of the Cu_3_SbSe_4_ compound via S alloying. Moreover, the development of TE materials with more cost-efficient constituent elements is of significant importance for large-scale practical applications.

Herein, we present the synthesis and thermoelectric characterization of the Cu_3_Sb(Se_1−*x*_S*_x_*)_4_ solid solutions with *x* covering the whole range from 0 to 1. The results demonstrate that the Cu_3_SbSe_4_-Cu_3_SbS_4_ solid solutions exhibit an extremely high Seebeck coefficient and ultralow thermal conductivity. Firstly, S alloying can widen the band gap, alleviating the bipolar effect. Additionally, S substitution in the lattice can significantly increase the carrier effective mass, leading to a remarkably high Seebeck coefficient of ~730 μVK^−1^. Moreover, the *κ*_lat_ can be significantly depressed owing to point defect scattering and Umklapp scattering, thus obtaining a minimum *κ*_lat_ of ~0.50 Wm^−1^K^−1^. Consequently, the multiple effects of S alloying boost the TE performance of the Cu_3_SbSe_4_-Cu_3_SbS_4_ solid solution, and a maximum ZT value of ~0.72 at 673 K is obtained for the Cu_3_SbSe_2.8_S_1.2_ sample.

## 2. Experimental Procedures

### 2.1. Synthesis

The Cu_3_Sb(Se_1−*x*_S*_x_*)_4_ solid solutions with varying S content (*x* = 0, 0.1, 0.2, 0.3, 0.4, 0.5, 0.6, 0.7, 0.8, and 1) were synthesized by vacuum melting and plasma-activated sintering (Ed-PAS Ⅲ, Elenix Ltd., Zama, Japan). Concretely, the synthesis was divided into two steps. The first step was to synthesize the primary powders. Firstly, the starting materials, consisting of high-purity components (Cu: 99.99 wt.%; Sb: 99.99 wt.%; Se: 99.999 wt.%; S: 99.99 wt.%) corresponding to the nominal composition of Cu_3_Sb(Se_1−*x*_S*_x_*)_4_ (*x* = 0–1), were carefully sealed in the quartz tube under high vacuum conditions (<10^−3^ Pa). Afterwards, the sealed tubes were incrementally heated to 1173 K with a controlled rate of 20 K/h and maintained at 1173 K for a duration of 12 h. Following a holding period, the tubes were cooled down with a relatively low rate of 10 K/h until reaching 773 K, and finally the samples were quenched into water. Subsequently, the acquired quenched ingots underwent direct annealing at 573 K for a period of 48 h to facilitate the uniformity of chemical compositions. After this step, the obtained ingots were finely pulverized using an agate mortar to produce uniform powders. The second step was to synthesize the target samples. The resultant powders were then introduced into a graphite die of Ø12.7 mm in diameter and treated using the PAS technique at 673 K for a duration of 5 min while applying an axial pressure of 50 MPa. In detail, the sintering temperature reached to 523 K after an activation time of 10 s under the activation voltage of 20 V and the activation current of 300 A, and then the current was manually adjusted to increase by a rate of 1.5 K/s to reach the desired sintering temperature of 673 K after 225 s; the temperature was then held for 300 s. Ultimately, the samples were furnace-cooled to room temperature.

### 2.2. Characterization

The X-ray diffraction (XRD) patterns for the Cu_3_Sb(Se_1−*x*_S*_x_*)_4_ (*x* = 0–1) solid solutions were conducted using a Bruker D8 advance instrument, which was equipped with Cu Kα radiation (λ = 1.5418 Å). Lattice parameters were refined using the Rietveld method, employing the HighScore Plus computer program for analysis. The morphologies and compositions of the afore-mentioned solid solutions were performed by a Nova NanoSEM450 (FESEM) and a JEM-2010F (HRTEM), equipped with a detector of energy-dispersive X-ray spectroscopy (EDS).

### 2.3. Thermoelectric Property Measurements

The as-sintered cylinders were processed into bars of 10 mm × 2 mm × 2 mm and disks of Ø12.7 mm × 2 mm. The bars were used for concurrently measuring *σ* and *S* by the commercial measuring system (LINSEIS, LSR-3) under a helium atmosphere, spanning a temperature range from room temperature to 673 K. Thermal conductivity was calculated using the equation of *κ* = *DC*_p_*ρ*. Herein, the *D*, *C*_p_, and *ρ* stand for the thermal diffusivity, specific heat, and density, respectively. The disks were used for simultaneously measuring D and C_p_ by utilizing a Laser Flash apparatus of Netzsch (LFA-457) under a static argon atmosphere. The *ρ* of the Cu_3_Sb(Se_1−*x*_S*_x_*)_4_ (*x* = 0–1) solid solutions were conducted using Archimedes’ methods. The relative densities, in relation to the theoretical density of 5.86 g cm^−3^, have been provided in Table S1. The *n* (carrier concentration) and *μ* (carrier mobility) of the afore-mentioned solid solutions at 300 K were performed using the Hall effect system (LAKE SHORE, 7707 A) according to the van der Pauw method under a magnetic field strength of 0.68 T.

## 3. Results and discussion

### 3.1. Crystal Structure

**Figure 1:**
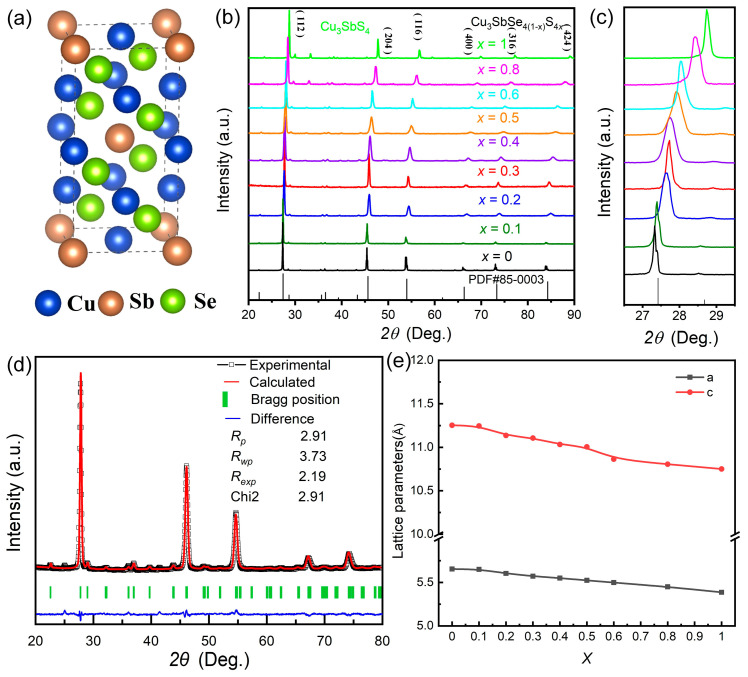
(**a**) The crystal structure of Cu_3_SbSe_4_; (**b**) X-ray diffraction (XRD) patterns and (**c**) magnified diffraction peaks corresponding to the (112) planes of Cu_3_Sb(Se_1−*x*_S*_x_*)_4_ (*x* = 0–1) samples; (**d**) Rietveld refinement profile of *x* = 0.3 solid solution; (**e**) Alterations in lattice parameters as S concentration varies.

The crystal structures and phase compositions for the Cu_3_Sb(Se_1−*x*_S*_x_*)_4_ (*x* = 0–1) samples were performed by XRD. Figure 1a shows the crystal structure of tetragonal Cu_3_SbSe_4_, with blue, gray, and green atoms representing Cu, Sb, and Se, respectively. As displayed in Figure 1b, the major diffraction peaks of the pristine sample (*x* = 0) are fully indexed to the zinc-blende-based tetragonal structure (*I-42m* space group) of Cu_3_SbSe_4_ (JCPDS No. 85-0003) without any detectable impurities [29]. With increasing S content (0 < *x* < 1), a continuous shift of the (112) diffraction peak towards higher angles can be seen (Figure 1c), demonstrating that S atoms replace Se at the Se site to form Cu_3_Sb(Se_1−*x*_S*_x_*)_4_ solid solutions. The shift in the diffraction peak can be ascribed to the smaller radius of S^2−^ (1.84 Å) in comparison to Se^2−^ (1.98 Å) [46]. For *x* = 1, the XRD peaks match the pattern of Cu_3_SbS_4_ (JCPDS No. 35-0581) [47].

The Rietveld refinement profiles of the Cu_3_Sb(Se_1−*x*_S*_x_*)_4_ (*x* = 0.3) samples based on the famatinite crystal structure are shown in Figure 1d. The data of the final agreement factors (*R_p_*, *R_wp_*, and *R_exp_*) of Cu_3_Sb(Se_1−*x*_S*_x_*)_4_ (*x* = 0–1) samples are listed in Table S2. The lattice parameter exhibits a linear decrease with increasing S concentration, and closely follows the expected Vegard’s law relationship [48] (Figure 1e), indicating the formation of Cu_3_SbSe_4_-Cu_3_SbS_4_ solid solutions.

### 3.2. Microstructure

**Figure 2:**
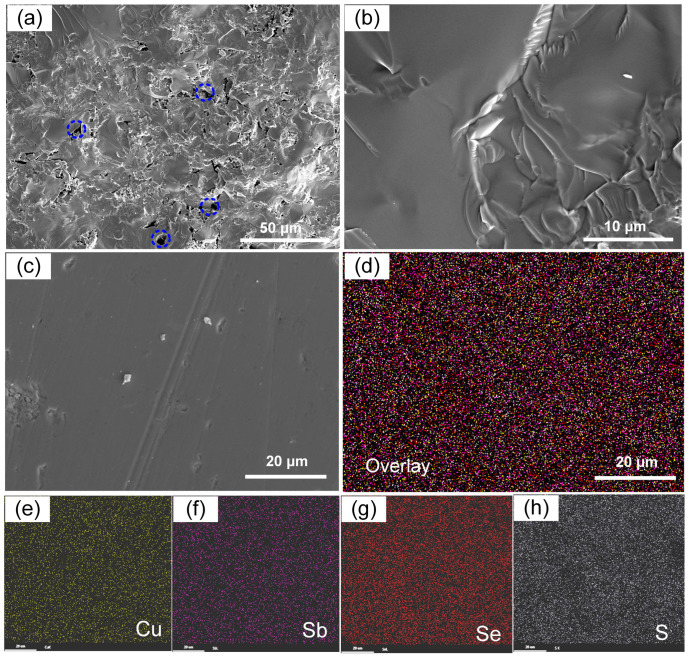
(**a**) SEM image of the fracture surfaces of the Cu_3_SbSe_2.8_S_1.2_ sample; (**b**) high magnification images of (**a**); (**c**) the corresponding EDS mapping for all constituent elements of selected region in (**b**); (**c**) SEM images of the polished surfaces of the Cu_3_SbSe_2.8_S_1.2_ sample; (**d**) The corresponding elemental mapping by EDS, obtained by overlaying the respective EDS signals directly arising from Cu (**e**), Sb (**f**), Se (**g**), and S (**h**).

The morphologies and chemical compositions of the Cu_3_Sb(Se_1−*x*_S*_x_*)_4_ (*x* = 0.3) sample were characterized by a SEM equipped with an EDS detector (Figure 2). As presented in Figure 2a,b, the SEM images of fracture surfaces (*x* = 0.3) indicated that they were isotropic materials. The nanopores (marked by the blue dotted circles) were observed on the fracture surface due to the Se/S volatilization of the synthesis process of the sample (Figure 2a), which can contribute to blocking the transport of mid-wavelength phonons [47]. To investigate the composition of the sample, we observed its polished surface (Figure 2c). According to the EDS elemental mapping (Figure 2d–h), the four constituent elements were uniformly distributed with no distinct micro-sized aggregations. This was combined with a back-scattered electron (BSE) image and elemental ratios (%), where Cu, Sb, Se, and S were present in proportions of 40.07:12.68:31.26:15.59 (as depicted in Figure S1), which demonstrated the formation of the Cu_3_SbSe_4_-Cu_3_SbS_4_ (*x* = 0.3) solid solution.

**Figure 3:**
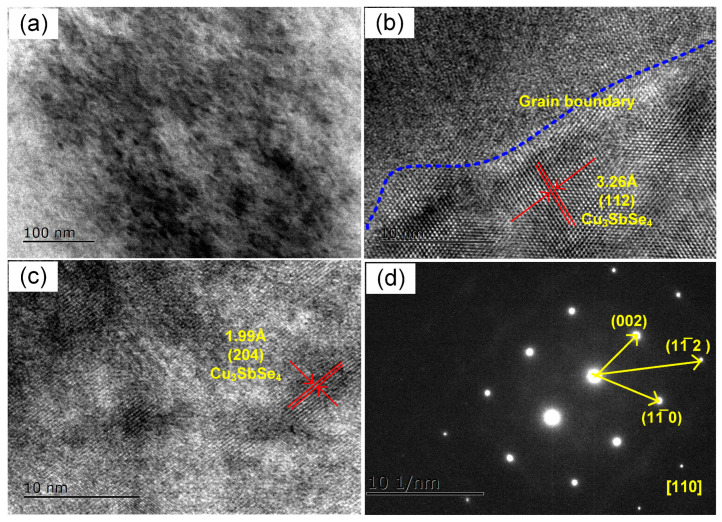
(**a**) The low-magnification image; (**b**,**c**) high-resolution TEM images; (**d**) SAED pattern taken from (**c**) of Cu_3_Sb(Se_1−*x*_S*_x_*)_4_ (*x* = 0.3) sample.

The morphologies and compositions of Cu_3_Sb(Se_1−*x*_S*_x_*)_4_ (*x* = 0.3) were further investigated at nanoscale using high-resolution TEM (HRTEM) (Figure 3). The TEM images demonstrated that many nanophases were distributed in the sample, and elemental mapping taking over the entire region revealed that the four constituent elements (Cu, Sb, Se, and S) were uniformly dispersed within the Cu_3_SbSe_4_-Cu_3_SbS_4_ solid solution (Figure 3a and Figure S2). As presented in Figure 3b, the grain boundary (indicated by blue dot lines) could be clearly observed in the sample. Meanwhile, as shown in Figure 3b,c, the crossed fringes, with interplanar spacing of 3.26 Å and 1.99 Å corresponded to the (112) and (204) planes of Cu_3_SbSe_4_, respectively [49]. Additionally, the SAED pattern taken from the Figure 3c along the [110] zone axis is displayed in Figure 3d. The ordered diffraction spots can be indexed to the (002), (1$\bar{1}$0), and (1$\bar{1}$2) planes of Cu_3_SbSe_4_, whose interplanar spacings are 5.64 Å, 4.06 Å, and 3.26 Å, respectively [50].

### 3.3. Charge Transport Properties

**Figure 4:**
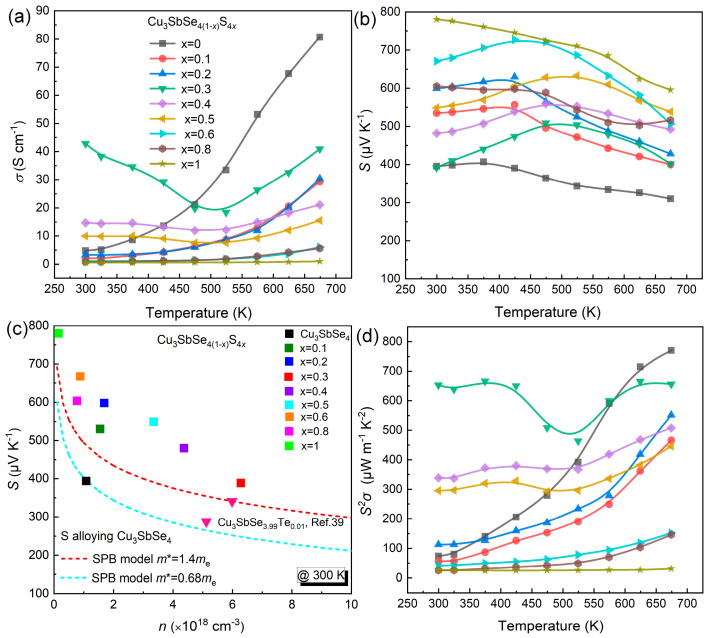
Temperature-dependent (**a**) electrical conductivity *σ*; (**b**) Seebeck coefficient *S*, the inset is *E*g; (**c**) Pisarenko relationship with m* in this work compared with other works at room temperature. The indigo and red broken line represent the Pisarenko relationship with m* ~ 0.68 and 1.4 *m*_e_, respectively. (**d**) power factor *S*^2^*σ* of Cu_3_Sb(Se_1−*x*_S*_x_*)_4_ (*x* = 0–1) samples.

To explore the effects of S alloying on the TE properties of the Cu_3_Sb(Se_1−*x*_S*_x_*)_4_ (*x* = 0–1) solid solutions, the charge transport properties were conducted. The temperature dependence of electrical conductivity (*σ*) of the Cu_3_Sb(Se_1−*x*_S*_x_*)_4_ (*x* = 0–1) solid solutions is displayed in Figure 4a. The pristine Cu_3_SbSe_4_ exhibited a monotonous increase in *σ* with rising temperature, demonstrating characteristic behavior of a non-degenerate semiconductor. For the *x* > 0.2 samples, the samples showed a transition from non-degenerate semiconductors to a partially degenerate regime [51]. The *σ* exhibited an initial decrease followed by an increase, with the minimum value occurring at ~473 K, indicating its association with bipolar conduction [38,52]. The *σ* of S alloying samples increased with the S contents until *x* = 0.3, after which it started to decrease with a higher S content. Notably, the *σ* improved from ~4.6 S/cm of pristine Cu_3_SbSe_4_ to ~42 S/cm of *x* = 0.3 solid solution at room temperature, arising from the augmented carrier concentration (Table S1). It is worth noting that the solid solutions with high S content (*x* > 0.5) had lower *σ* compared to the pristine Cu_3_SbSe_4_, which was ascribed to the reduced *n* (carrier concentration) and diminished *μ* (carrier mobility). Furthermore, due to the intensified lattice vibration at elevated temperatures, the solid solutions exhibited lower *σ* than the pristine sample at high temperatures, indicating that the intensified lattice vibration in the solid solutions at elevated temperatures hindered the carrier migration [40,53].

Figure 4b illustrates the temperature-dependent *S* of the Cu_3_Sb(Se_1−*x*_S*_x_*)_4_ (*x* = 0–1) samples. The p-type semiconductor behavior of solid solutions, characterized by dominant hole carriers, was evidenced by the positive S value observed across the entire temperature range.

Notably, the *S* value of the samples exhibited an initial ascent followed by a subsequent descent as the temperature rose, ultimately reaching its zenith at ~473 K. This behavior can be attributed to the influence of the bipolar effect [54]. The maximum *S* of ~730 μVK^−1^ was obtained from the *x* = 0.6 solid solution. We calculated the *E*_g_ of the Cu_3_Sb(Se_1−*x*_S*_x_*)_4_ (*x* = 0–1) samples with the formula: *E*_g_ = 2*eS*_max_*T*, where *E*_g_, *e*, *S*_max_, and *T* represent the band gap, elementary charge, maximal Seebeck coefficient, and the associated temperature, respectively [55]. The calculated *E*_g_ for the pristine Cu_3_SbSe_4_ of ~0.30 eV aligned well with the reported literature [36,56]; the results are displayed in Figure S3. Consequently, the introduction of alloyed S played a role in enlarging *E*_g_ from ~0.30 eV to ~0.69 eV, thus widening the band gap to alleviate the bipolar effect. For the semiconductors, we note that the increase in *S* (|*S*|) was directly proportional to the carrier effective mass and *n*^−2/3^. We calculated the Pisarenko relation between |*S*| and *n* (indigo and red dashed lines with m* ~ 0.68 and 1.4 *m*_e_, respectively) based on the single parabolic band model (SPB), as follows [57,58]:

(1) $$S=\frac{8\pi^{2}k_{B}^{2}}{3e\hbar^{2}}m*T{\left(\frac{\pi }{3n}\right)}^{2/3}$$

where *k_B_*, ℏ represent the Boltzmann constant, and Planck constant, respectively. The calculated m* was significantly enhanced from 0.68 for pristine Cu_3_SbSe_4_ to 5.03 *m*_e_ for the *x* = 0.6 sample (Table S1). As seen in Figure 4c, the calculated m* based on *S* (experimental values) of Cu_3_Sb(Se_1−*x*_S*_x_*)_4_ (*x* = 0.1–1) samples were above the Pisarenko line. Furthermore, the m* depended directly on the *E*_g_ ($\frac{\hbar^{2}k_{B}^{2}}{2m*}=Ε\left(1+\frac{Ε}{{Ε}_{g}}\right)$), where *E* the energy of electron states), which deviated from a single Kane band model [21,59,60], thus confirming the large *S* was related to *E*_g_ and m*. Consequently, the decreased carrier concentration (*x* > 0.5) and increased m*, resulted in the significant enhancement of *S*.

The temperature dependence of the power factors (*S*^2^*σ*) of the Cu_3_Sb(Se_1−*x*_S*_x_*)_4_ (*x* = 0–1) samples are presented in Figure 4d. The *S*^2^*σ* of Cu_3_SbSe_4_-Cu_3_SbS_4_ solid solutions exhibited a similar temperature-dependent behavior as the electrical conductivity (*σ*). The temperature-dependent trend observed in the *S*^2^*σ* was mirrored in the behavior of the *σ* for the Cu_3_SbSe_4_-Cu_3_SbS_4_ solid solutions. Owing to their relatively elevated σ and S values, these samples demonstrated larger *S*^2^*σ* values compared to the pristine Cu_3_SbSe_4_, particularly within the lower temperature range. Notably, the *x* = 0.3 sample achieved a larger *S*^2^*σ* value than the other samples, and the peak *S*^2^*σ* value of Cu_3_SbSe_2.8_S_1.2_ sample was ~670 μW m^−1^ K^−2^ at 673 K.

### 3.4. Thermal Transport Properties

**Figure 5:**
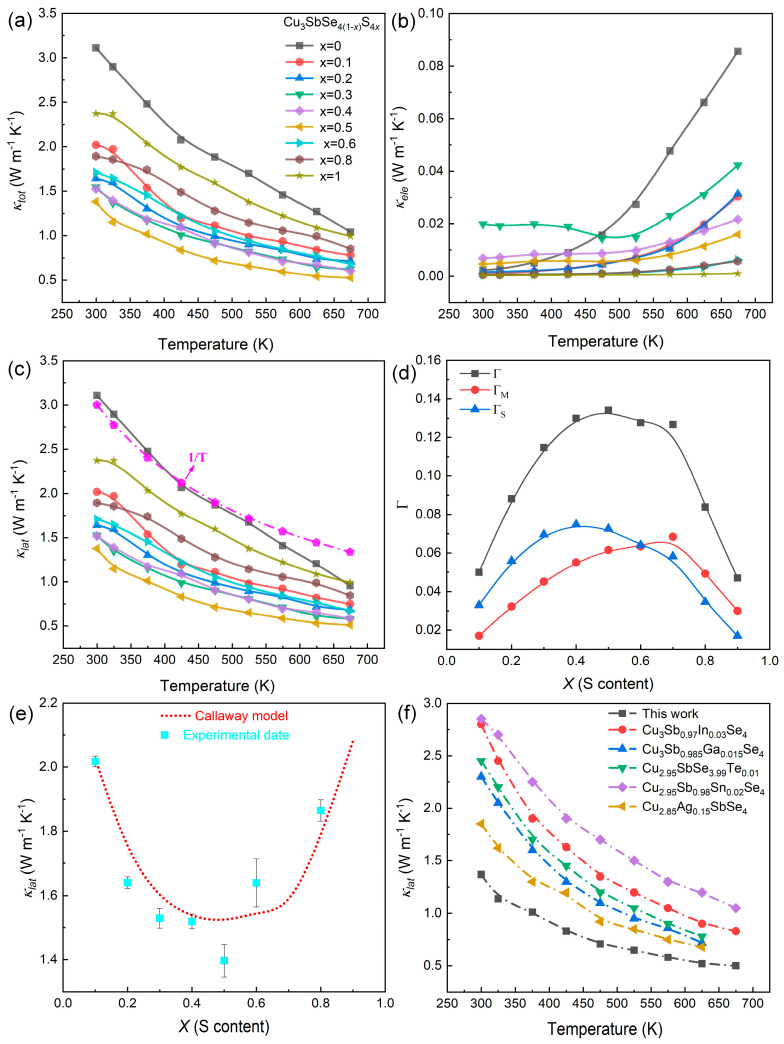
Temperature-dependent (**a**) total thermal conductivity *κ*_tot_; (**b**) electronic thermal conductivity *κ*_ele_; (**c**) lattice thermal conductivity *κ*_lat_ of Cu_3_Sb(Se_1−*x*_S*_x_*)_4_ (*x* = 0–1) samples, and (**d**) imperfection scaling parameters; (**e**) *κ*_lat_ at 300 K, the red dotted lines is calculated by the Callaway model; (**f**) the comparison of *κ*_lat_ of Cu_3_SbSe_4_-based materials [33,34,36,38,39].

The temperature dependence of the thermal transport properties of the Cu_3_Sb(Se_1−*x*_S*_x_*)_4_ (*x* = 0–1) solid solutions are presented in Figure 5 and Figure S4. Obviously, the *κ*_tot_ decreased with increasing temperature, mainly attributed to the increased scattering by lattice vibrations at elevated temperatures [8,40,53] (Figure 5a). For instance, the *κ*_tot_ of pristine Cu_3_SbSe_4_ decreased from ~3.11 Wm^−1^K^−1^ at 300 K to ~1.03 Wm^−1^K^−1^ at 673 K. Similarly, the *κ*_tot_ of the *x* = 0.5 sample decreased from ~1.37 Wm^−1^K^−1^ at 300 K to ~0.52 Wm^−1^K^−1^ at 673 K. Generally, the *κ*_lat_ can be obtained by subtracting the electronic part (*κ*_ele_) from the *κ*_tot_ using the Wiedeman–Franz relationship (the details are displayed in Supplementary Material) [61,62]:

(2) $$\kappa {\;}_{\mathrm{ele}}=L\sigma \;Τ$$

where *L* is the Lorenz number and it can be expressed as Equation (3) [63,64]:

(3) $$L=1.5+\exp\;\left[\frac{-\left|S\right|}{116}\right]$$

The calculated *L* values of the Cu_3_Sb(Se_1−*x*_S*_x_*)_4_ (*x* = 0–1) samples ranged from 1.5 to 1.6 W Ω K^−2^, and the results are listed in Figure S5. Owing to the enhanced carrier concentration (*x* < 0.6), the *κ*_ele_ showed a slight increase at low temperature after S alloying, as described in Figure 5b. The *κ*_lat_ of the Cu_3_Sb(Se_1−*x*_S*_x_*)_4_ (*x* = 0–1) samples is plotted in Figure 5c, indicating a significant decrease within the measured temperature range after S alloying.

To explore the effects of S alloying on the phonon scattering and the significant reduction in *κ*_lat_, the *κ*_lat_ of the Cu_3_Sb(Se_1−*x*_S*_x_*)_4_ (*x* = 0–1) compounds was evaluated at room temperature by the Debye–Callaway model. The primary scattering mechanisms under consideration were point defect scattering and Umklapp scattering. Then, the *κ*_lat_ of the pristine ($\kappa_{lat}^{pristine}$) and S-alloyed (*κ*_lat_) Cu_3_SbSe_4_ compounds could be computed based on the Debye–Callaway model [48,65,66]:

(4) $$\frac{\kappa_{\mathrm{lat}}^{}}{\kappa_{\mathrm{lat}}^{\mathrm{pristine}}}=\frac{\arctan\left(u\right)}{u},\;u^{2}=\frac{\pi^{2}\theta_{D}\Omega }{hv^{2}}\kappa_{\mathrm{lat}}^{\mathrm{pristine}}\Gamma$$

where *u*, *θ*_D_, Ω, *h*, and *ν* represent the scaling parameter, Debye temperature, volume per atom, Planck constant, and average speed of sound, respectively (herein, *θ*_D_ = 131 K and *ν*= 1991.2 m/s [46]). Γ is the imperfection scale parameter, which is associated with the Γ_m_ (mass fluctuation) and Γ_s_ (strain field fluctuation) [67]:

(5) $$\Gamma =\Gamma_{m}+\Gamma_{s}=x(1-x)\left[{\left(\frac{\Delta M}{M}\right)}^{2}+\varepsilon {\left(\frac{\Delta r}{r}\right)}^{2}\right]$$

where *x*, ∆*M*/*M* and ∆*r*/*r* are the S concentration in one molecular, the relative change of atomic mass, and atomic radius owing to the replacement of Se with S, respectively. The *ε* value can be computed using the following formula [68]:

(6) $$\varepsilon =\frac{2}{9}{\left(\frac{6.4\gamma \left(1+\upsilon_{p}\right)}{1-\upsilon_{p}}\right)}^{2}$$

where, *γ* and *υ*_p_ are the Grüneisen parameter and Poisson ratio, respectively (here, *γ* = 1.3 [46] and *υ*_p_ = 0.35 [69]).

The values of Γ_m_ and Γ_s_ for the Cu_3_Sb(Se_1−*x*_S*_x_*)_4_ compounds are presented in Table S3. Figure 5d shows how the scattering parameters Γ_m_ and Γ_s_ changed with varying Se-alloying levels. It was observed that Γ_m_ was smaller than Γ_s_ when the S content *x* ≤ 0.6, indicating that the Γ_s_ (strain field fluctuation) contributed greatly to the drop of *κ*_lat_. As for the *x* > 0.6 samples, the Γ_m_ (mass fluctuation) was the dominant. It is commonly accepted that the atomic radius of S is different from that of the Se atom, inducing a localized lattice distortion and leading to local field fluctuations that hinder the propagation of heat-carrying phonons [70,71]. However, with increasing S content, the mass fluctuation gradually became the dominant factor. The experimental *κ*_lat_ closely aligned with the curve calculated by the Callaway model (Figure 5e), suggesting that point defects made a great contribution to suppress the *κ*_lat_ in the Cu_3_Sb(Se_1−*x*_S*_x_*)_4_ solid solutions [48]. For a more comprehensive evaluation of our results, a comparison of the our *κ*_lat_ data with the recently reported values of Cu_3_SbSe_4_ are illustrated in Figure 5f [33,34,36,38,39]. Remarkably, the Cu_3_Sb(Se_1−*x*_S*_x_*)_4_ (*x* = 0.5) sample achieved an outstandingly low *κ*_lat_ of ~0.50 W m^−1^ K^−1^ at 673 K.

### 3.5. Figure of Merit (ZT)

**Figure 6:**
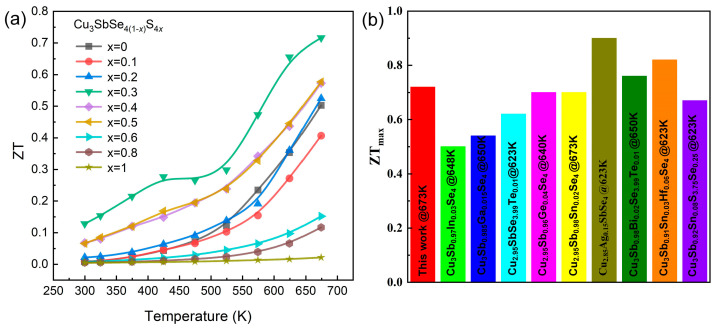
(**a**) Temperature-dependent figure of merit (ZT); (**b**) Comparison of ZT_max_ of Cu_3_SbSe_4_-based materials [33,34,35,36,37,38,39,40,72].

The temperature-dependent ZT of the Cu_3_Sb(Se_1−_*_x_*S*_x_*)_4_ (*x* = 0–1) samples are illustrated in Figure 6a. With the benefit of the collaborative enhancement of electrical and thermal transport properties, the x = 0.3 sample attained a maximum ZT value of ~0.72 at 673 K, which was 44% higher than that of pristine Cu_3_SbSe_4_. To further analyze our TE properties, the comparison of the ZT_max_ of the Cu_3_SbSe_4_-based materials is given in Figure 6b [33,34,35,36,37,38,39,40,72]. Obviously, our ZT_max_ of 0.72 was higher than that of other Cu_3_SbSe_4_-based materials, such as Cu_3_Sb_0.97_In_0.03_Se_4_ ~0.5, Cu_3_Sb_0.985_Ga_0.015_Se_4_ ~0.54, Cu_3_SbSe_3.99_Te_0.01_ ~0.62, Cu_3_Sb_0.92_Sn_0.08_S_3.75_Se_0.25_ ~0.67, Cu_2.95_Sb_0.96_Ge_0.04_Se_4_ ~0.70, and Cu_2.95_Sb_0.98_Sn_0.02_Se_4_ ~0.7 and is comparable to the ZT values for Cu_3_Sb_0.98_Bi_0.02_Se_3.99_Te_0.01_ ~0.76 and Cu_3_Sb_0.91_Sn_0.03_Hf_0.06_Se_4_ ~0.76. Although the Cu_3_Sb(Se_1−_*_x_*S*_x_*)_4_ (*x* = 0–1) samples had relative low ZT values in comparison with other high-performance TE materials, further enhancements of the ZT values can potentially be achieved by tuning the carrier concentration, dual-incorporation, and/or introducing band engineering.

## 4. Conclusions

In summary, a series of Cu_3_SbSe_4_-Cu_3_SbS_4_ solid solutions were synthesized by vacuum melting and plasma-activated sintering (PAS) techniques, and the effects of S alloying on TE performance were investigated. S alloying can widen the band gap, effectively alleviating the bipolar effect. Additionally, the *S* (Seebeck coefficient) was significantly improved because of the increased m*. Furthermore, the substitution of S for Se in Cu_3_SbSe_4_ lattice led to noticeable local distortions, yielding large strain and mass fluctuations to suppress the *κ*_lat,_ thus decreasing the *κ*_lat_ and *κ*_tot_ to ~0.50 Wm^−1^K^−1^ and ~0.52 Wm^−1^K^−1^ at 673 K, respectively. Consequently, a peak ZT value of ~0.72 was obtained at 673 K for the Cu_3_Sb(Se_1−*x*_S*_x_*)_4_ (*x* = 0.3) sample. Based on these results, it is speculated that further improvement in the figure of merit of Cu_3_Sb(Se_1−*x*_S*_x_*)_4_ solid solutions can be obtained by enhanced electrical transport properties. Our research offers a new strategy to develop high-performance TE materials in solid solutions via elemental alloying.

## Data Availability

Data will be made available on reasonable request.

## Supplementary Materials

The supporting information can be downloaded at: https://www.mdpi.com/article/10.3390/nano13192730/s1.
