# Supplementary material for: Realizing the Ultralow Lattice Thermal Conductivity of Cu3SbSe4 Compound via Sulfur Alloying Effect
Source: Nanomaterials (Basel). 2023 Oct 8;13(19):2730. doi: 10.3390/nano13192730 (PMC10574639; doi:10.3390/nano13192730)
Supplement: Supplementary file 1 [file nanomaterials-13-02730-s001.zip › nanomaterials-2636141-supplementary.pdf]

Supplementary materials for

Realizing the Ultralow Lattice Thermal Conductivity of Cu_3SbSe_4 compound via Sulfur Alloying Effect

Lijun Zhao[1], Haiwei Han[1], Zhengping Lu[1], Jian Yang[2,*], Xinmeng Wu[1], Bangzhi Ge[3], Lihua Yu[1], Zhongqi Shi[4], Abdulnasser M. Karami[5], Songtao Dong[1], Shahid Hussain[2], Guanjun Qiao[2], and Junhua Xu[1,*]

[1]School of Materials Science and Engineering, Jiangsu University of Science and Technology, Zhenjiang 212100, China

[2]School of Materials Science and Engineering, Jiangsu University, Zhenjiang 212013, China

[3]State Key Laboratory of Solidification Processing, and Key Laboratory of Radiation Detection Materials and Devices, Ministry of Industry and Information Technology, Northwestern Polytechnical University, Xi'an 710072, China

[4]State Key Laboratory for Mechanical Behavior of Materials, Xi'an Jiaotong University, Xi'an 710049, China

[5]Department of Chemistry, College of Science, King Saud University, Riyadh 11451, Saudi Arabia

**Corresponding authors:*

jyyangj@ujs.edu.cn (J. Yang);

jhxu@just.edu.cn (J.H. Xu)

Theoretical methods

1. Calculations of Lorenz number (*L*) and electrical thermal conductivity (*κ_{ele}*)

Measured *D* for pristine Cu_3SbSe_4 and $Cu_3Sb(Se_{1-x}S_x)_4$ (x=0.1–1) samples are shown in Figure S4. The lattice thermal conductivity (κ_{lat}) is obtained by subtracting electrical thermal conductivity (κ_{ele}) from κ_{tot} using Wiedeman-Franz relationship [1].

$$\kappa_{ele} = L\sigma T \tag{S1}$$

where *L* is the Lorenz number and it can be expressed as equation (2) [2,3].

$$L = 1.5 + \exp\left[\frac{-|S|}{116}\right] \tag{S2}$$

The estimated Lorenz number of $Cu_3Sb(Se_{1-x}S_x)_4$ (x=0–1) samples are listed in Figure S5.

Supporting figures

1. SEM, BSE and EDS spectrum of $Cu_3Sb(Se_{1-x}S_x)_4$ (x=0.3) sample

The four constituent elements Cu, Sb, Se and S are uniformly distributed in the selected area (marked A), and the atomic ratio (%) of Cu, Sb, Se and S is 40.07: 12.68: 31.26: 15.59 (Figure S1c), demonstrating that the formation of a solid solution of Cu_3SbSe_4-Cu_3SbS_4 (x=0.3), which is consistent with XRD results.

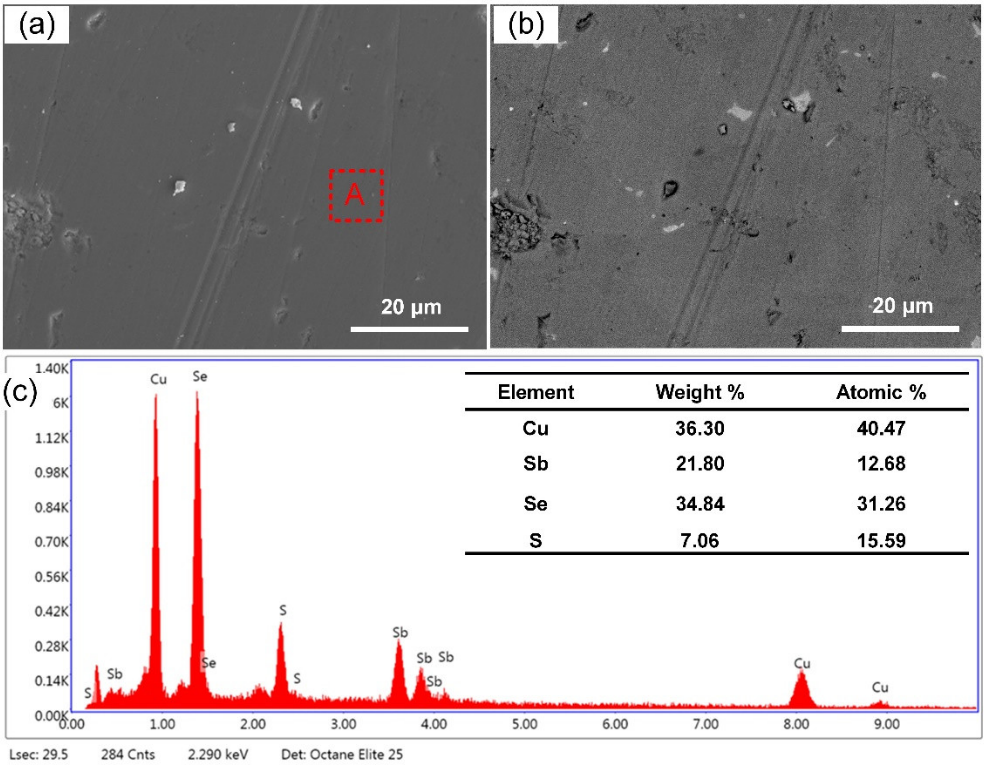

Figure S1 (a) SEM, (b) BSE and (c) EDS spectrum of polished surface of $CuSbSe_{2.8}S_{1.2}$ sample.

2. TEM morphology and EDS mapping images of $Cu_3Sb(Se_{1-x}S_x)_4$ (x=0.3) sample.

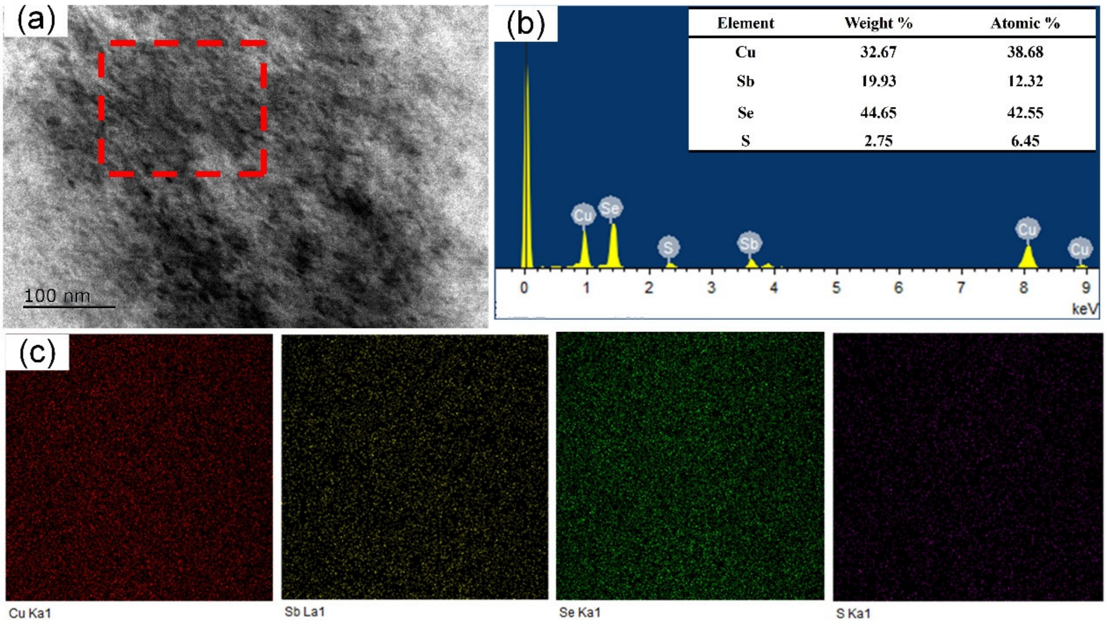

Figure S2 TEM morphology images of $Cu_3SbSe_{2.8}S_{1.2}$ sample.

3. Band gap (E_g) of $Cu_3Sb(Se_{1-x}S_x)_4$ samples

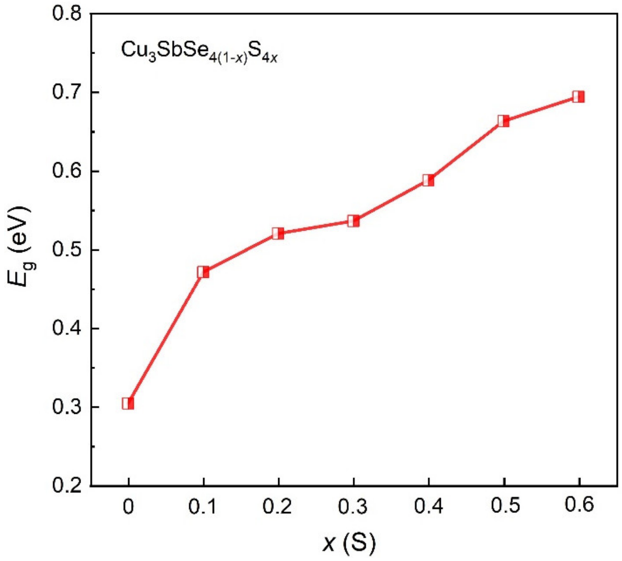

Figure S3 Band gap (E_g) of $Cu_3Sb(Se_{1-x}S_x)_4$ (x=0–0.6) samples.

4. Measured *D*

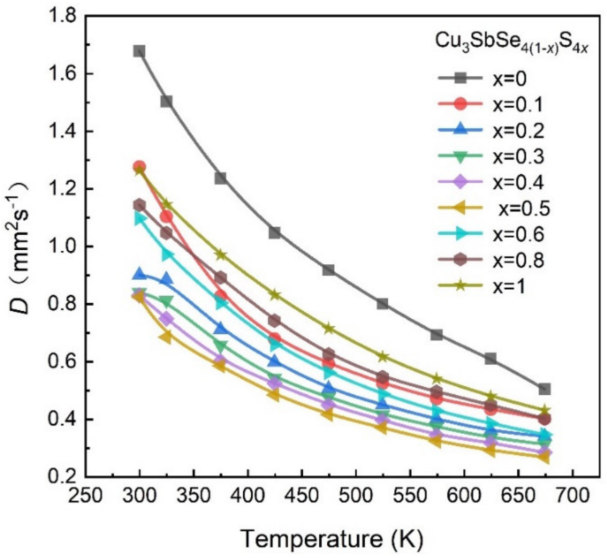

Figure S4 Temperature-dependent *D* of $Cu_3Sb(Se_{1-x}S_x)_4$ (x=0–1) samples.

5. Calculated *L*

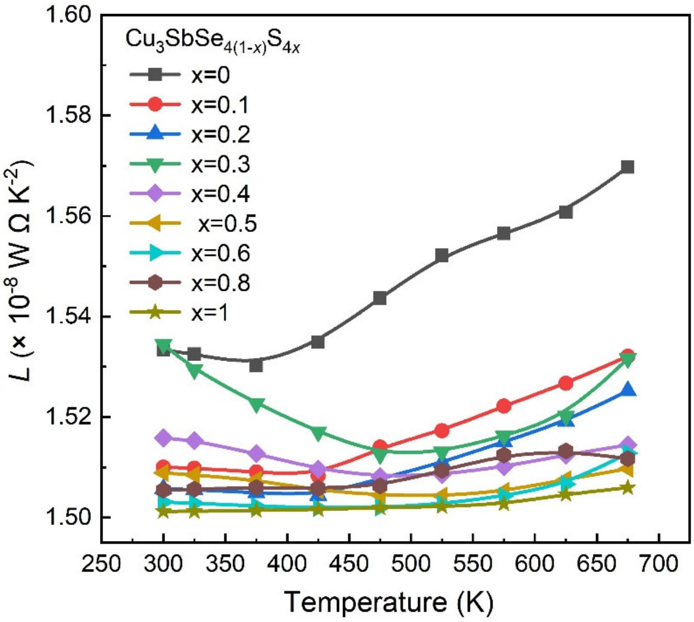

Figure S5 Temperature-dependent Lorenz number (*L*) of $Cu_3Sb(Se_{1-x}S_x)_4$ (x=0–1) samples.

Supporting table

Table S1 Relative density, carrier concentration, Hall mobility and carrier effective mass for $Cu_3SbSe_{4(1-x)}S_{4x}$(x=0–1) samples at room temperature (300 K).

Samples	Density（gcm^{-3})	Relative density (%)	Carrier concentration (10^{18} cm^{-3})	Hall mobility (cm^2 V^{-1} s^{-1})	Carrier effective mass (m_e)
x=0	5.79	98.8	1.08	62.2	0.68
x=0.1	5.53	94.3	1.55	24.7	2.48
x=0.2	5.39	92.0	1.69	26.2	4.45
x=0.3	5.33	91.0	6.28	48.6	2.12
x=0.4	4.9	83.6	4.37	37.5	3.36
x=0.5	4.72	80.5	3.35	35.2	4.77
x=0.6	4.65	79.4	0.88	30.4	5.03
x=0.8	4.56	77.8	0.77	15.1	2.78
x=1			0.15	12.5	3.62

Table S2 The Refined parameters and quality factors of $Cu_3SbSe_{4(1-x)}S_{4x}$ (x=0–1) samples.

Nominal composition	Space group	a (Å)	c (Å)	V (Å^3)	R_p (%)	R_{wp} (%)	R_{exp} (%)	*Chi2*
x=0	$I\bar{4}2m$	5.6537	11.252	359.66	5.97	8.06	2.35	11.8
x=0.1	$I\bar{4}2m$	5.6505	11.246	359.06	5.39	7.18	2.34	9.43
x=0.2	$I\bar{4}2m$	5.6035	11.136	349.66	6.47	8.77	4.82	3.11
x=0.3	$I\bar{4}2m$	5.5718	11.106	344.78	2.91	3.73	2.19	2.91
x=0.4	$I\bar{4}2m$	5.5491	11.033	339.73	5.77	7.40	4.64	2.55
x=0.5	$I\bar{4}2m$	5.5238	11.005	335.79	5.77	7.32	4.62	2.51
x=0.6	$I\bar{4}2m$	5.4980	10.863	328.37	7.43	9.80	4.69	4.37
x=0.8	$I\bar{4}2m$	5.4492	10.806	320.87	6.64	8.58	4.82	3.17
x=1	$I\bar{4}2m$	5.3866	10.750	311.92	7.69	9.78	4.57	4.59

Table S3 Parameters of $Cu_3SbSe_{4(1-x)}S_{4x}$(x=0–1) samples obtained by fitting the experimental lattice thermal conductivity data to the Debye-Callaway model using v=1991.2m/s, and θ_D =131K, respectively [4–6] .

Nominal composition	Γ_m	Γ_s	Γ	u	κ_{lat} (W m^{-1} K^{-1})	κ_{exp} (W m^{-1} K^{-1})
x=0	0	0	0	—	3.0	—
x=0.1	0.0171	0.0427	0.0597	1.5468	1.9335	2.015
x=0.2	0.0323	0.0724	0.1047	2.0420	1.6387	1.636
x=0.3	0.0452	0.0901	0.1353	2.3146	1.5074	1.521
x=0.4	0.0552	0.0969	0.1521	2.4465	1.4504	1.514
x=0.5	0.0616	0.0940	0.1556	2.4674	1.4417	1.373
x=0.6	0.0635	0.0831	0.1466	2.3879	1.4752	1.707
x=0.7	0.0684	0.0755	0.1438	2.3580	1.4882	—
x=0.8	0.0492	0.0449	0.0941	1.9014	1.7144	1.880
x=0.9	0.0300	0.0220	0.0520	1.4093	2.0301	—
x=1	0	0	0	—	—	—

References

[1] Mahan, G.; Bartkowiak, M. Wiedemann–Franz law at boundaries. Appl. Phys. Lett. **1999**, 74, 953–954.

[2] Kim, H.S.; Gibbs, Z.M.; Tang, Y.G.; Wang, H.; Snyder, G.J. Characterization of Lorenz number with Seebeck coefficient measurement. APL. Mater. **2015**, 3, 041506.

[3] Wang, B.Y.; Zheng, S.Q.; Wang, Q.; Li, Z.L.; Li, J.; Zhang, Z.P.; Wu, Y.; Zhu, B.S.; Wang, S.Y.; Chen, Y.X.; Chen, L.Q.; Chen, Z.-G. Synergistic modulation of power factor and thermal conductivity in Cu_3SbSe_4 towards high thermoelectric performance. Nano Energy **2020**, 71, 104658.

[4] Skoug, E.J.; Cain, J.D.; Morelli, D.T. High thermoelectric figure of merit in the Cu_3SbSe_4-Cu_3SbS_4 solid solution. Appl. Phys. Lett. **2011**, 98, 261911.

[5] Yang, J.; Meisner, G.P.; Chen, L.D. Strain field fluctuation effects on lattice thermal conductivity of ZrNiSn-based thermoelectric compounds. Appl. Phys. Lett. **2004**, 85, 1140−1142.

[6] Wan, C.L.; Pan, W.; Xu, Q.; Qin, Y.X.; Wang, J.D.; Qu, Z.X.; Fang, M.H. Effect of point defects on the thermal transport properties of $(La_xGd_{1-x})_2Zr_2O_7$: Experiment and theoretical model. Phys. Rev. B **2006**, 74, 144109.
